# How do studies assess the preventability of readmissions? A systematic review with narrative synthesis

**DOI:** 10.1186/s12874-019-0766-0

**Published:** 2019-06-19

**Authors:** Eva-Linda Kneepkens, Corline Brouwers, Richelle Glory Singotani, Martine C. de Bruijne, Fatma Karapinar-Çarkit

**Affiliations:** 1 Department of Clinical Pharmacy, OLVG Hospital, Jan Tooropstraat 164, 1061 AE Amsterdam, The Netherlands; 2 Department of Public and Occupational Health, Amsterdam UMC, Vrije Universiteit Amsterdam, Amsterdam Public Health Research Institute, Van der Boechorststraat 7, NL-1081 BT Amsterdam, The Netherlands

**Keywords:** Hospital readmission, Avoidability, Preventability, Assessment, Review, Patient interview

## Abstract

**Background:**

A large number of articles examined the preventability rate of readmissions, but comparison and interpretability of these preventability rates is complicated due to the large heterogeneity of methods that were used.

To compare (the implications of) the different methods used to assess the preventability of readmissions by means of medical record review.

**Methods:**

A literature search was conducted in PUBMED and EMBASE using “readmission” and “avoidability” or “preventability” as key terms. A consensus-based narrative data synthesis was performed to compare and discuss the different methods.

**Results:**

Abstracts of 2504 unique citations were screened resulting in 48 full text articles which were included in the final analysis. Synthesis led to the identification of a set of important variables on which the studies differed considerably (type of readmissions, sources of information, definition of preventability, cause classification and reviewer process). In 69% of the studies the cause classification and preventability assessment were integrated; meaning specific causes were predefined as preventable or not preventable. The reviewers were most often medical specialist (67%), and 27% of the studies added interview as a source of information.

**Conclusion:**

A consensus-based standardised approach to assess preventability of readmission is warranted to reduce the unwanted bias in preventability rates. Patient-related and integrated care related factors are potentially underreported in readmission studies.

**Electronic supplementary material:**

The online version of this article (10.1186/s12874-019-0766-0) contains supplementary material, which is available to authorized users.

## Background

The general goal of hospital care is to restore the patient’s health condition to the pre-admission state or to discharge the patient in the best possible health condition. Nevertheless, approximately 20% of the hospital admissions in the US result in an unplanned readmission within 30 days after discharge, of which a subset is preventable [1]. These readmissions result in an increase in cost, workload for caregivers and a potential health risk for patients [2]. Hence, hospital readmission rates are increasingly being used to monitor quality improvement and cost control [3]. Currently, hospitals are being benchmarked in several countries based on their readmissions rate. In some of these countries, high rates can result in financial penalties and they are used as a policy to stimulate hospitals to implement improvement plans [4].

These improvement plans are generally complex and costly, therefore, prediction models to identify patients who are at risk for readmissions are being developed [5]. However, these models are often not validated prospectively or in other datasets [6]. Furthermore, electronic prediction algorithms tend to overestimate potentially preventable readmissions [7]. It is important to understand the complex mechanism behind readmissions and to achieve an accurate prediction of preventable readmissions. This can be achieved through medical record review, preferably combined with narratives obtained from patient interviews [7], and other sources, such as a general practitioner (GP).

Many studies have examined the preventability rate of readmissions, but comparison and interpretability of these preventability rates are complicated by the large heterogeneity of methods used to assess the preventability [8]. In addition, (systematic) reviews that studied the preventability of readmissions did not focus on *the method of assessment*, and whether specific methodological options affect the likelihood of finding a high or low preventability rate [7, 9–11]. Understanding the implications of different methodological options could aid in solving a piece of the readmission puzzle. Therefore, the objective of this study is to compare methods and discuss all studies in which preventability of hospital readmissions was assessed by use of medical record review. By these means, we hope to provide the reader guidance in how to conduct and report their study data on readmissions.

## Methods

### Data source and searches

A systematic literature search was applied in Pubmed and Embase in December 2016. In the first step of the search strategy(MeSH and tiab)-terms for “readmission” and “rehospitalization” were combined with terms such as “avoidability” or “preventability” (see Additional file 1). In the next step this search was combined with terms such as “quality of health care”, “quality indicators”, and “chart review”. In the last step conference abstracts were excluded from the search. For this search a medical information specialist was consulted. All citations were imported into Endnote X 7.3.1TM.

### Study selection

A stepwise study selection (described below) was conducted using a consensus-based approach. In case of disagreement, an independent senior researcher was consulted (FKC and MdB).

- *Step 1*: Two researchers (CB, EK) independently screened all abstracts using the major inclusion and exclusion criteria, i.e. English language, manual assessment using, at least, the medical record and a clear method description regarding preventability assessment in the aim, method or result section, see Additional file 2. Cohen’s kappa for interrater agreement (CB and EK) was good (k = 0.70).
- *Step 2*: References of included articles were assessed and a cited reference search in Web of Science and Scopus (CB and EK) was performed additionally for all full text articles included in step 1 (*n* = 77).
- *Step 3*: Detailed inclusion and exclusion criteria (Additional file 2) were applied to all 77 articles by two researchers independently (equally divided over CB, EK, RS). This additional step was conducted to ensure that the finally selected articles were able to help us reach our study objective; 1. Full text article in English; 2. The article should be based on original patient data; in case of ≥2 or more papers used the same, or partly the same, patient sample only the paper with the most thoroughly described methodology of preventability assessment was included; 3. Studying hospital readmissions should be clearly stated in the aim/ primary objective; 4. Duration between index and readmission should be ≤6 months; 5. Assessment of preventability should be performed via manual medical record review or at least, it should be clear that the preventability assessment was performed on an individual patient level by a care provider and/or trained researcher which cannot be performed without a review; 6. The methodology of the preventability assessment of readmissions should be described clearly in order to perform data-synthesis; this includes a description of criteria of preventability and/or a cause classification (≥3 cause categories) of preventable readmissions and the reviewer process (at least 2 independent reviewers and disagreement should have been solved by reaching consensus and/ or a third independent reviewer OR, in case not performed/ nor reported (NR) > 50 medical files of readmitted patients should have been reviewed).

### Critical appraisal of individual sources of evidence

A validated critical appraisal was performed to evaluate the reliability, value and relevance of each article. Commonly used quality appraisal tools were not suitable because of the large heterogeneity in study designs. Hence, a critical appraisal tool was used which is developed by the Cochrane recommendations for narrative data synthesis and analysis [12]. This critical appraisal was implemented in the data synthesis. The goal of using the narrative synthesis is, similar to other appraisal tools, to avoid bias. The process of narrative data synthesis is rigorous and transparent, in which the process is specified in advance. These process steps were followed systematically.

### Data synthesis

A (textual) narrative synthesis was performed to compare the methods of the included studies and this led to the identification of a set of important variables. The following variables were systematically collected and described in the *Result section*: study design characteristics, sources of information to assess preventability, definition of preventability, cause classification (classifying the cause of a readmission) and reproducibility (i.e. the reviewer process and training) (see Additional file 3).

There are several important considerations to take into account prior to reading the results; (1) the cause classification and preventability assessment are often integrated; meaning specific causes were predefined as always preventable or not preventable. These studies were called a priori preventability cause classifications; (2) some articles reported the number and percentage of *readmissions* while others reported the number of *readmitted patients,* or both. For the purpose of this article, we reported the percentage of preventable readmissions/readmitted patients based on the actual number of reviewed files within one month (if this could be extracted from the provided data); (3) cause classification refers to description of at least three causes; (4) lastly, the index admission is the admission prior to readmission.

### Data extraction and analysis

Data was collected (CB, EK, RS) using a predefined form which included study characteristics and relevant data with regard to the method of preventability assessment. During the preliminary data synthesis, all data extracted by one researcher was checked by at least one other researcher (CB, EK, RS). During the systematic approach a double check or consensus-discussion was only performed in case of doubt because all definitions were thoroughly discussed after the preliminary phase. Lastly, potential associations between preventability rates and study characteristics were explored using the independent sample t test, Mann-Whitney u test or χ2 test depending on the variable distribution. A value of < 0.05 was considered to be statistically significant. The data were analysed with SPSS version 21.0 software (IBM, New York, USA).

## Results

**Fig. 1:**
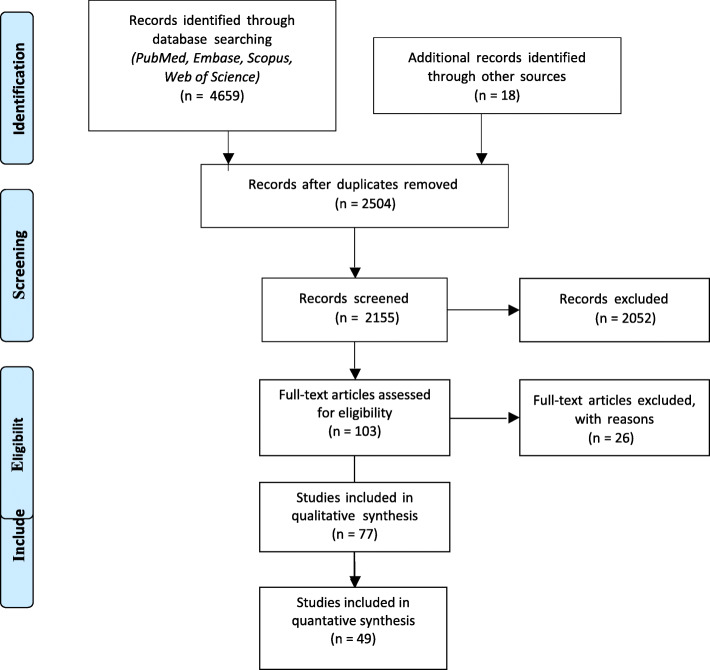
PRISMA 2009 Flow Diagram

Abstracts of 2504 unique citations were screened resulting in 77 full text articles that reported on the assessment of preventability. Step 3 of the stepwise study selection resulted in the final inclusion of 48 (64%) articles. The other studies (*n* = 29) were excluded because the primary objective of the paper was not focussed on readmissions, the duration (discharge index admission to readmission) was longer than 6 months, or because the readmission method of preventability assessment was not explicitly described in the method section. A minimal dataset for the excluded articles, and the reason for exclusion, is shown in Additional file 4. An overview of the selection process is shown in Fig. 1*.*

### Study design and characteristics

As shown in Table 1, the studies were published between 1988 and 2017, often as single center studies (*n* = 37; 77.1%) and often performed in the USA (*n* = 32; 66.6%). Twelve studies focused on a specific diagnosis (*n* = 12) or a group (e.g. elderly or children) within a single department (e.g. internal medicine). Furthermore, nine studies examined all-cause readmissions, meaning that patients readmitted at all departments were eligible for inclusion [13–21]. Additional file 5 provides more detailed information on the descriptive characteristics of the studies.

**Table 1:** Descriptives of included studies

Study characteristics (n = 48)	No. or percentage of studies
Year of publication, range	1988–2016
Country, n (%)	
USA	32 (67%)
Other	16 (33%)
Study design, n (%)	
Retrospective	30 (63%)
Cross-sectional	10 (21%)
Prospective	8 (16%)
Setting, n (%)	
Single center	37 (77%)
Multicenter	11 (23%)
Number of readmissions reviewed, n ± SD	226 ± 208
Planned readmission excluded, n (%)	
Yes	30 (63%)
No	11 (23%)
Not reported	7 (14%)
All-cause readmission, n (%)	
Yes	9 (19%)
No	39 (81%)
Percentage preventable readmissions, mean, ± SD	27,8 ± 16,7%
Scoring of preventability, n (%)	
Binary	22 (46%)
Scale	4 (8%)
Categorical	17 (35%)
Not applicable (a priori studies)	5 (11%)
A priori preventable causes determined, n (%)	
Yes	32 (67%)
No	16 (33%)
Training of reviewers, n (%)	
Yes	16 (33%)
No	2 (4%)
Not reported	30 (63%)
Number of reviewers, n (%)	
Individual	8 (16%)
Duo	23 (48%)
Duo + team	2 (4%)
Individual + team	2 (4%)
Team	5 (11%)
Individual or duo + panel	3 (6%)
Other	5 (11%)
Double check, n (%)	
All cases	28 (58%)
Partially	7 (15%)
No	3 (6%)
Not reported	10 (21%)
Additional sources, n (%)	
Interview or survey	13 (27%)
None	35 (73%)

### Sources of information

Thirteen articles (*n* = 13) used additional sources of information, such as interviews, questionnaires or surveys, in addition to the manual medical record review, see Table 1 [14, 21–32]. Additional file 6 provides more information on the interviews with care providers and/or patients. In 7 studies the patient was approached [21–23, 25, 30–32] and in 5 studies the patient or caregiver was approached [14, 26–29]. In 4 studies it was mentioned that the results of the interview were available for the reviewers during their assessment of preventability, however, it was not specified if and how these results influenced the preventability assessment [14, 22, 26, 29]. In the paper of Toomey et al. [27] the preventability was first assessed without the interview results. Subsequently, the interview results were shared with the reviewer and it was documented how this additional information changed the review outcome. This resulted in new information in 31.2% of the cases and a change in the final preventability score in 11.8%. However, no further details were published regarding which information of the interview was crucial for the reviewer to change his or her opinion. The other 5 studies did not specify whether or not the additional patient/caregiver information was used to assess the preventability [25, 28, 30–32]. In the study of Burke et al. [23], only 6 patients were interviewed during a pilot phase. After the pilot, they concluded that the interviews did not provide additional data to the patient’s medical record.

Six studies interviewed at least one care provider, of which mostly the GP, see Additional file 6. Four studies reported that the results of the care provider interview were available for the reviewers [14, 22], or were included in the preventability judgement [26] and one reported that the opinion of the interviewee was included in the final preventability judgement via equal weighing of their opinion with the opinion of the audit team [24].

### Preventability

A subset of articles used a very broad definition of preventability, such as the study of Ryan et al.;

*‘Providers were given no specific guidelines for deciding whether a readmission was preventable. This allowed use of their different backgrounds in choosing which elements of the clinical record to focus on.*’ [33]

In addition, the majority of the articles did not explicitly provide the definition of preventability, instead they often directly referred to the cause classification (see Additional file 7), such as Williams et al.; *‘It was noted that readmission could have been avoided if more effective action had been taken in one or more of five areas: preparation for and timing of discharge, attention to the needs of the carer, timely and adequate information to the general practitioner and subsequent action by the general practitioner, sufficient and prompt nursing and social services support, and management of medication.’ *[28]

### Cause classification

The cause classification (the description of at least three causes) that was used by the studies varied largely. Several studies used an existing tool, like the STate Action on Avoidable Rehospitalizations (STAAR) initiative [14, 21, 27, 30, 34] or root cause approach [5, 18, 24, 35–37] but all others adapted an existing tool or developed their own tool based on previous publications. For the purpose of this article we focused only on the distinction between studies using an a priori preventability cause classification [13–16, 19, 21–26, 31, 35, 37–55], or not [5, 17, 18, 20, 27–30, 32, 33, 36, 56–59], see Table 2*.* As an example of an a priori cause classification, Clarke et al. reported, Unavoidable causes: chronic or relapsing disorder; unavoidable complication, readmission for social or psychological reason, reasons probably beyond control of hospital services, completely different diagnosis from previous admission. Avoidable causes: recurrence or continuation of disorder leading to first admission, recognised avoidable complication, readmission for social or psychological reason, reasons probably within control of hospital services. [39]

**Table 2:** Preventability assessment of the included studies (N = 48)

Author	Planned read-missions excluded?^a^	No. read-missions reviewed^b^	No. of preventable unplanned readmissions	% preventable unplanned readmissions^c^	Scoring of preventability	A priori preventable causes determined	Training of reviewers	Reviewers^d^	Double check of preventability	Additional sources used for the review
Agrawal	yes	30	11	36,7	categorical	yes	no	individual	no	–
Auerbach	yes	1000	269	26,9	scale	yes	yes	duo	all cases	Interview^f^
Balla	yes	271	90	33,2	binary	no	NR	duo	all cases	Interview^e^
Bianco	no	229	100	43,7	binary	yes	yes	duo	all cases	Interview^e^
Burke	yes	335	78	23,3	categorical	yes	yes	duo	all cases	Interview^e^
Cakir	NR	85	4	4,7	categorical	yes	NR	individual	NR	–
Clarke	yes	74	18,9	25,5	categorical	yes	NR	duo or team	all cases	–
Dawes	yes	258	55	21,3	categorical	yes	yes	duo + team	All cases	–
Epstein	no	50	1	2,0	categorical	yes	NR	duo + team	All cases	–
Feigenbaum	no	537	250	46,6	categorical	yes	yes	duo	all cases	Interview^f^
Fluitman	yes	50	26	52,0	binary	yes	NR	duo	all cases	–
Frankl	yes	318	28	8,8	categorical	yes	NR	individual + team	partially	–
Gautam	yes	109	16	14,7	binary	yes	NR	individual + team	NR	Interview^f^
Glass	NR	96	25	26,0	binary	no	NR	NR	NR	–
Greenberg	yes	97	22	22,7	categorical	yes	NR	duo	NR	–
Hain	no	200	40	20,0	scale	yes	yes	panel	all cases	–
Halfon	yes	429	40	9,3	NA	yes	NR	duo	partially	–
Harhay	yes	201	19	9,5	binary	yes	yes	duo	all cases	
Jiminez-Puente	no	185	44	23,9	binary	yes	NR	duo	all cases	–
Jonas	no	248	15	6,0	binary	yes	NR	individual + panel	partially	–
Kelly	yes	32	22	68,8	binary	yes	NR	duo	all cases	
Koekkoek	no	298	45	15,1	categorical	no	yes	individual	NR	–
Maurer	yes	32	3	9,4	binary	yes	NR	duo	partially	–
Meisenberg	yes	72	22	30,6	binary	yes	NR	duo	all cases	–
Miles	yes	437	24	5,5	categorical	no	NR	duo	partially	–
Mittal	yes	35	15	42,9	binary	no	NR	duo	all cases	
Nahab	no	174	92	52,9	NA	yes	NR	duo	all cases	–
Nijhawan	yes	130	62	47,7	NA	yes	NR	duo + panel	all cases	–
Njeim	NR	161	51	31,7	binary	no	yes	individual	no	–
Oddone	NR	514	183	34,2*	categorical	no	yes	duo	partially	–
Pace	yes	140	19	13,9	categorical	yes	NR	duo	all cases	–
Ryan	yes	40	NR	26,7	categorical	no	yes	team	all cases	–
Saunders	yes	282	51	18,1	binary	yes	NR	team	all cases	–
Shah	no	407	149	36,6	NA	yes	NR	duo	all cases	–
Shalchi	NR	63	45	71,4	binary	no	NR	team	all cases	–
Shimizu	no	153	50	32,7	binary	yes	NR	panel	all cases	Interview^e^
Stein	yes	213	64	29,5	binary	no	NR	individual	NA	Interview^f^
Sutherland	yes	47	11	23,4	NA	no	NR	individual	NR	Interview^e^
Tejedor-Sojo	no	147	62	42,2	categorical	yes	yes	team	NR	–
Toomey	yes	305	90	29,5	scale	no	yes	team	all cases	Interview^f^
Vachon	yes	98	14	14,3	binary	yes	NR	individual	NR	–
Van Walraven	yes	317	70	22,1	scale	no	yes	duo	partially	Interview^e^
Vinson	NR	66	10	15,2	categorical	no	NR	duo	NR	Interview^e^
Wallace	yes	204	41	20,1	binary	yes	yes	duo	all cases	–
Wasfy	NR	893	380	42,6	categorical	yes	yes	duo	all cases	–
Weinberg	yes	50	3	6,0	binary	yes	NR	panel	all cases	–
Williams	yes	133	78	58,6	binary	no	NR	individual	no	Interview^f^
Yam	yes	603	246	40,8	binary	no	no	Duo + panel	all cases	–

^a^Planned readmissions were considered excluded when the planned readmissions were excluded before preventability was assessed.

^b^Number of reviewed cases is based on the number of included patients for whom preventability of a readmission was assessed, based on the number of included readmissions for which preventability was assessed, or based on the number of preventability assessments performed.

^c^In case a study calculated the percentage of preventable readmissions for multiple time durations (time between index and readmission) the time duration of 30 days (or closest to 30 days) was chosen to increase the comparability of the results with the other studies. *Based on phase 2 of the study

^d^individual = a single reviewer independently assessed the preventability of the readmission without a double check by other reviewers or a consensus meeting; individual + team/panel = a single reviewer independently assessed the preventability of the readmission, but a double check is performed on a selection of cases; duo = both reviewers assessed the preventability of the readmissions and came to a mutual agreement; duo + team/panel = both reviewers assed the preventability added by a team or panel which could advise the two reviewers in case a mutual agreement on the preventability was not achieved; team or panel: cases are directly reviewed by a team of 3 to 4 persons.

^e^Interview (or questionnaire or survey) was conducted with the patient only;

^f^Interview (or questionnaire or survey) was conducted with the patient and the care provider (general practitioner of physician).

The majority of the studies did not report whether they assessed the causal relationship (i.e. whether the readmission is related to the care provided during index admission) explicitly, but ‘causative or causal’ could be extracted from the cause and/or preventability criteria [15, 16, 23, 32, 43, 44, 52, 53]. In addition, a few articles included information on ‘related readmissions’. These readmissions were defined as related based on the same diagnosis (or complication), the same department, or medical/clinically related [13, 20, 35, 37, 38, 40, 42, 48–51, 56, 57]. Another term used was ‘causation’ [18, 27, 32].

### Reproducibility/reviewer process

As shown in Table 2, the number of reviewers varied between 1 and 35. Four studies had ≥10 reviewers [17, 32, 36, 43]. The reviewers were most often physicians (specialists) or a combination hereof [5, 13, 15–18, 20–23, 25, 27, 28, 30–32, 35–39, 41, 42, 45, 47, 50, 51, 53–55, 57, 58]. A subset of studies included a multidisciplinary study team consisting of physicians, general practitioners, a medical officer, case managers, (specialized) nurses, medical record specialists, social workers and/or administrative staff [14, 24, 29, 33, 44, 46, 48]. In three studies senior residents performed the review supervised by a senior physicians [19, 26, 59]. In five studies no information on expertise was reported [40, 49, 52, 56, 60].

As shown in Table 2 roughly three options for review were possible: a single reviewer without a double check [13, 17, 28, 38, 51, 59], a single reviewer double checked by a second reviewer [15, 18, 32, 36, 45] or a team [24, 40, 43] or a team of 3 to 4 persons which reviewed the readmissions directly [20, 25, 27, 33, 41, 49, 54]. Agreement and consensus regarding the preventability was handled differently: a double review of each readmission was performed meaning that both reviewers assessed the preventability of the readmissions and came to a mutual agreement [14, 16, 18, 19, 22, 23, 29–31, 35, 42, 44, 46, 47, 50, 52, 53, 57]. In some cases a team or panel was consulted when mutual agreement on the preventability was not achieved [5, 48, 55, 60]. Two studies could not be allocated to one of these review categories because the review process was not clearly described or because they used a mix of different methods [39, 56].

A subset of the included articles offered some kind of support to the reviewers to clarify and solidify classification criteria, to increase the uniformity between the assessments or to refine the study logistics and/or survey instrument or implemented as an educational program [59]. The support was mainly provided by means of a training, instruction session, pilot [17, 22, 27, 32, 36, 42, 52] and/or discussion of preventable causes and readmissions [14, 16, 18, 27, 36, 37, 42, 52, 53, 55]; other options were: a study protocol or review guide [22, 37, 40], a bimonthly meeting and/or an educational program [59].

Agreement was calculated in different ways: the interrater agreement (i.e. kappa coefficient) [15, 16, 23, 30, 40, 42, 50, 52, 53, 60], intrarater reliability [49] or both [36]; other options were the interclass correlation and a concordance coefficient [39, 41] or the percentage of agreement on preventability [25, 33, 37, 43, 48, 55]. A low level of agreement was associated with the presence of multiple conditions; the more difficult it was to disentangle the reason for readmissions, the higher the chance of disagreement between the reviewers [39].

## Discussion

The aim of this study was to compare the currently available methods to assess the preventability of readmissions, and the implications of these methods in terms of the preventability rates that were found. The focus on the methodology of preventability assessment is unique to this review and the results can be used to contribute to the development of a consensus-based approach to assess the preventability of readmissions. Furthermore, we aimed to provide the reader guidance in how to design, conduct and report their study in a well-considered manner.

A large heterogeneity in study designs was identified which limits the comparability of the preventability rates. In addition, it is currently not possible to distinguish which part of the variation in preventability rate really represents variation in quality of care. Only a consensus-based standardised approach to assess preventability can reduce the unwanted bias caused by methodological differences and contextual factors.

The interpretation of the results was further complicated by inconsistent use of important study definitions (i.e. definition of preventability). Studies were also contradictory, for example some studies regarded patient factors such as noncompliance as a potential preventable cause for readmissions as others regarded this non-preventable.

Most studies used an a priori preventability cause classification approach which is less time-consuming to apply. An a priori approach is comparable with an electronic algorithm to predict potentially preventable readmissions. In these cases a prediction is based on a specific connections between variables (i.e. matching or correlated admission diagnosis codes). Such predictive algorithms, based on administrative data, are increasingly used. However, the performance (in terms of the discriminative ability) of risk predictive models has varied significantly [61]. Although, manually applying these algorithm rules may improve the likelihood of identifying true potentially preventable readmissions, it still does not invite the reviewer to look beyond the predefined potential causes of preventability. On the other hand, performing chart review is time-intensive and has a limited reproducibility. Our results show that researchers try to optimize the reproducibility in different ways, e.g. the training of reviewers, a double check with the use of a second reviewer and/or a (multidisciplinary) team. Nevertheless, these different variables were not significantly associated with preventability percentages.

In the majority of studies the preventability assessment was performed by a physician or several physicians (often from the same department or specialty). This might increase the risk of reluctancy to consider alternatives to one’s preferred line of thought (i.e. potential causes related to other specialties). In addition, many patients are treated by multiple care providers and this might complicate optimal assessment of the readmissions when a single (medical specialty) perspective is used [62]. It is currently unknown which readmissions should be reviewed by a multidisciplinary team and how that would affect the preventability outcome and the causes found.

Most studies only assessed preventability based on chart review. However, charts usually do not contain all the potential information that can influence the preventability assessment, for example information on the collaboration between care providers or lack of social support. Future research should therefore focus more on examining which information (i.e. on communication, follow-up care or information needs) from which care providers is valuable to optimize the preventability assessment [22]. The studies that did obtain additional information from the patient- and primary care provider perspective often did not describe the added value of this information. This is a missed opportunity because collecting this information is often complex and time consuming.

The use of readmission rates to benchmark hospital performance is controversial [11]. Readmissions often seem to be caused by a multitude of causes, some of which are not modifiable by the hospital (i.e. home environment or social support), meaning hospitals are penalized for causes that are beyond their control. In addition, the use of readmission as a quality indicator may provide a wrong incentive, for example by lengthening hospital stays to decrease the chance of readmissions or hesitation to readmit a patient who might benefit from it. This is contradictory to what the indicator was designed for, namely to provide the incentive to provide higher quality care. Hence, readmissions do not seem to be a useful indicator of quality of care [3].

This was the first review which compared the different methods used to assess preventability of unplanned hospital readmissions via medical record review, however, some limitations need to be discussed. Unfortunately, the heterogeneity of the studies was large, therefore, the options for a quality appraisal tool were limited and a meta-analysis was not possible. To compensate for this, we performed a (textual) narrative synthesis based on the Cochrane recommendations [12]. In addition, since there was no uniformity amongst studies on the use of (key)words in their title and abstract, it could be that some studies on readmissions were missed during our search because these terms were not included in our search strategy. All phases were either consensus based –driven and/or performed by at least two independent data extractors. However, this procedure could not prevent that some amount of interpretation bias was present during data collection, synthesis and the interpretation.

In conclusion, many articles on preventability of readmissions are currently available, however, a meaningful comparison is limited due to the large study heterogeneity (i.e. the included population, definition inconsistencies and variation in methods to assess preventability). Moreover, the majority of assessments was based on a hospital and physician perspective only, resulting in a potentially underestimation of factors related to coordination of care (e.g. integrated care), patient or social support system. Readmissions are most likely multifactorial and readmission rate reduction is a shared responsibility within the network of care providers and the patient or carer himself. Therefore, the scope should switch from the hospital to the organization of care within the region and patient participation. Overall, we recommend that researchers carefully consider the different methodological options (i.e. study population, setting and its modifiable factors, and type of resources) prior to initiating a study to assess the preventability of readmissions. In Table 3 we outlined a few important methodological aspects of readmission studies and provided the advantages, disadvantages and recommendations for each of these aspects. Furthermore, we recommend for future research that the methodological considerations of each readmission study are explicitly reported to increase reproducibility and comparability (e.g. the number of reviewers, review process).

**Table 3:** Advantages, limitations and considerations of several study design options

	Advantage	Limitation	Recommendations
Single center versus multicenter	Single center studies provide information on one’s own performance which is needed to induce a quality improvement cycle	For scientific purposes it is easier to identify which results can be extrapolated to other institutes when the results are obtained via a multicenter study. Furthermore, in a multicenter study benchmarking between the centers is possible.	Compare the results with the current literature on the preventability of readmissions, and be aware of (inter)national and regional differences in organization of care.
Population (Focus on a specific population versus a broad population)	Manual review is easier to perform on a specific group (e.g. diagnosis heart failure or department).	Focus on single group can cause underestimation of the preventability readmission rate and/or underreporting of certain causes.	Consider a multidisciplinary panel or team to review the readmissions to reduce blind spots.
Relatedness (focus on readmissions that are related to the index readmission versus all-cause readmissions)	Readmissions related to the index hospitalization will generally identify causes that are related to hospital care.	All-cause readmissions are easier to identify based on administrative data, provide a broad scope and will identify other causes; for example causes related to care in the primary care setting.	Determine the scope of the quality improvement cycle; to identify causes related to hospital care or to care of a region
Type of readmissions (unplanned versus planned readmissions)	Selecting only unplanned readmissions resembles the readmissions that are used to calculate the readmission quality indicator	Planned readmission might also have preventable causes which will be missed if planned readmissions are excluded	Determine whether you consider unplanned readmissions preventable prior to starting a readmission study
Setting and sources (focus on hospital versus an integrated care network)	Assessment based on a hospital’s perspective only requires the medical record as single source.	Fragmented and incomplete description of the patient’s journey can result in underreporting causes related to integrated care, patient and social factors.	Interview, questionnaire or survey a (subset) of patients and or primary care providers.
Information and sources (which sources and information to include; and in which order)	Including the full medical record, outpatient data and even additional sources (e.g. interviews) can change the perspective on preventability and its causes.	Reviewers might use a different approach of obtaining/using the (additional) information which can create unwanted differences in the perspective on preventability. Note that for an interview of stakeholders a cross-sectional or prospective study design is needed to reduce recall bias.	A strict protocol and logbook as well as training prior to start of the study. Consider to provide additional information stepwise to assess its added value on the preventability assessment.
A priori (preventability) cause classification	Easier to perform and probably better agreement between reviewers.	Does not invite reviewer to look beyond this list of predefined (potentially preventable) causes and can therefore narrow the reviewer’s view.	Usa a multidisciplinary approach with more than one reviewer. The use of a strict protocol and logbook as well as training prior to start of the study, and case discussion during the study, can increase uniformity
Reviewers (single reviewer versus duo/team)	Using a single reviewer to perform the preventability assessment is less time-consuming.	Due to the poor reproducibility some kind of double check is needed.	Double (partial) review can increase uniformity. If a double check is not possible, consider a team or panel discussion (of a subset) of cases. Moreover, case discussion adds to the learning and awareness component of the medical record review process.
Experience	Residents as reviewer can contribute to the learning environment.	Some studies suggest that years of experience can influence the preventability assessment.	Approach seniors to be available for supervision, double check by a senior and/or training, strict protocol or discussion meetings.
Complete or partial double check	A partial double check is less time consuming.	This can influence the agreement calculation.	In case of partial double check use the appropriate analysis.
Final preventability judgment (binary score versus scale or category)	Using a binary score for preventability is straightforward and easy to interpret	Since the majority of readmissions have multifactorial causes a binary preventability score does not resemble reality; a scale of category offers the option of making a thoughtful decision	Use a scale or category which includes intermediate scores on preventability. Be clear on which categories are used/combined to calculate the preventability percentage.

## Additional files

Additional file 1: Search strategy. (DOCX 14 kb)

Additional file 2: Inclusion criteria. (DOCX 85 kb)

Additional file 3: Definition of variables. (DOCX 18 kb)

Additional file 4: Characteristics of studies which were excluded based on the inclusion criteria of the flow chart (N=29). (DOCX 24 kb)

Additional file 5: Detailed descriptives of included studies (N=48). (DOCX 33 kb)

Additional file 6: Details regarding patient (and/or caregiver) interview and care provider interview. (DOCX 40 kb)

Additional file 7: Definition of preventability. (DOCX 24 kb)

## Data Availability

The datasets supporting the conclusions of this article are included within the article.

## Abbreviations

GP: General practitioner
NR: Not reported
STAAR: The STate Action on Avoidable Rehospitalizations
