# Supplementary material for: How do studies assess the preventability of readmissions? A systematic review with narrative synthesis
Source: BMC Med Res Methodol. 2019 Jun 19;19:128. doi: 10.1186/s12874-019-0766-0 (PMC6585018; doi:10.1186/s12874-019-0766-0)
Supplement: Supplementary file 1 — Search strategy. (DOCX 14 kb) [file 12874_2019_766_MOESM1_ESM.docx]

**Additional file 1: Pubmed search strategy**

(((("Patient readmission"[MeSH Terms] OR readmi*[tiab] OR re-admi*[tiab] OR rehospitali*[tiab] OR re-hospitali*[tiab])) AND (Avoid*[tiab] OR prevent*[tiab] OR unnecessar*[tiab] OR needless OR inevitab*[tiab] OR inappropriate*[tiab] OR accountab*[tiab]))) AND ((("Quality of Health Care"[Mesh:NoExp] OR "Quality Indicators, Health Care"[Mesh])) OR (Medical record*[tiab] OR health record*[tiab] OR quality indicat*[tiab] OR quality of healthcare[tiab] OR quality of health care[tiab] OR quality of care[tiab] OR manual review*[tiab] OR chart review*[tiab] OR chart note*[tiab] OR chart search*[tiab] OR case review*[tiab] OR case note*[tiab] OR interview*[tiab] OR perspective*[tiab] OR survey*[tiab] OR checklist*[tiab] OR reviewer*[tiab] OR consensus[tiab] OR agreement[tiab] OR root cause anal*[tiab]))

**Hits: 1038**

**Embase search strategy**

#2 'hospital readmission'/exp OR 'hospital readmission' OR readmi*:ab,ti OR 're admi*':ab,ti OR rehospitali*:ab,ti OR 're hospitali*':ab,ti AND (avoid*:ab,ti OR prevent*:ab,ti OR unnecessar*:ab,ti OR needless:ab,ti OR inevitab*:ab,ti OR inappropriate*:ab,ti OR accountab*:ab,ti)

#6 ((medical OR health) NEXT/1 record*):ab,ti OR (quality NEAR/2 indicat*):ab,ti OR 'quality of healthcare':ab,ti OR 'quality of health care':ab,ti OR 'quality of care':ab,ti OR ((manual OR chart OR case) NEXT/1 review*):ab,ti AND (chart NEXT/1 (note* OR search*)):ab,ti OR (case NEXT/1 note*):ab,ti OR interview*:ab,ti OR perspective*:ab,ti OR survey*:ab,ti OR checklist*:ab,ti OR reviewer*:ab,ti OR consensus:ab,ti OR agreement:ab,ti OR ('root cause' NEXT/1 anal*):ab,ti

#14 'health care quality'/de OR 'health care survey'/de OR 'incident report'/de OR 'medical error'/exp OR 'consensus development'/de OR 'root cause analysis'/de OR 'outcomes research'/de

#15 #6 OR #14

#18 #2 AND #15 NOT ([conference abstract]/lim OR [conference paper]/lim OR [conference review]/lim)

**Hits: 962 citations**
