# Supplementary material for: How do studies assess the preventability of readmissions? A systematic review with narrative synthesis
Source: BMC Med Res Methodol. 2019 Jun 19;19:128. doi: 10.1186/s12874-019-0766-0 (PMC6585018; doi:10.1186/s12874-019-0766-0)
Supplement: Supplementary file 2 — Inclusion criteria. (DOCX 85 kb) [file 12874_2019_766_MOESM2_ESM.docx]

Additional file **2: Inclusion criteria**

**PRIMARY INCLUSION CRITERIA**

Is paper written in English? ^1^

Is paper based on original data? ^2^

Is the primary objective of the paper focussed on (unplanned) hospital readmissions? ^3^

Is the paper based on readmissions with a duration (discharge index admission to readmission) ≤6 months, or if not, was the causal relationship assessed between index admission and readmission? ^4^

Assessment of preventability based on medical chart review or, in case of survey/ interview etc., based on individual patient data?^5^

Was the method of preventability assessment explicitly described in the method section? ^6^

Did ≥2 reviewers assess preventability? ^7^

Were the medical files of >50 readmitted patients/readmissions reviewed? ^8^

Were the healthcare-related causes (≥3) of potentially preventable readmissions discussed in the result section? ^9^

Yes

No

Exclusion

Yes

No

Exclusion

Yes

No

Exclusion

No/ NR

Exclusion

Yes

No

Exclusion

Yes

Yes

Inclusion

No

No/ NR

Yes

No

Exclusion

Yes

Yes

**SECONDARY INCLUSION CRITERIA**

No

**Detailed description of inclusion and exclusion criteria**

Criteria in figure are organized hierarchical order.

1. Full text article in English.
2. The paper should be based on original patient data; in case of ≥2 or more papers used the same, or partly the same, patient sample only the paper with the most thoroughly described methodology of preventability assessment was included.
3. Studying hospital readmissions should be clearly stated in the aim/ primary objective of the study.
4. Duration between index and readmission should be *≤6* months or >6, if the causal relationship between index admission and readmission was assessed and described; to prevent inclusion of non-related readmissions and maintain uniformity between the studies.
5. Assessment of preventability should be performed via manual medical record review or at least, it should be clear that preventability assessment was performed on individual patient level by a care provider and/or trained researcher which cannot be performed without a review.
   1. NOT: General themes of causes of preventable readmissions. Administrative databases or electronic algorithms. Manual review of a sample only to validate an electronic algorithm is excluded.
6. The methodology of preventability assessment *of readmissions* should be described clearly in order to be reproducible; This includes a description of criteria of preventability and/or a cause classification (*≥3* healthcare-related cause categories) of preventable readmissions and the reviewer process. If a paper does not fulfill the previous criteria it should at least fulfill the following criteria:
   1. Assessment of *preventability* is performed by at least 2 independent reviewers and disagreement should have been solved by reaching consensus and/ or a third independent reviewer OR, in case not performed/ nor reported (NR) >50 medical files of readmitted patients should have been reviewed.
   2. AND, the healthcare-related causes (≥3) of *preventable readmissions* should be described in the result section.
   3. NOT: only an overall percentage of preventable readmissions is reported without any form of subdivision in cause categories.
7. Although, not excluded, intervention studies are not the focus of this review.
