# Supplementary material for: How do studies assess the preventability of readmissions? A systematic review with narrative synthesis
Source: BMC Med Res Methodol. 2019 Jun 19;19:128. doi: 10.1186/s12874-019-0766-0 (PMC6585018; doi:10.1186/s12874-019-0766-0)
Supplement: Supplementary file 3 — Definition of variables. (DOCX 18 kb) [file 12874_2019_766_MOESM3_ESM.docx]

Additional file **3: Definition of variables**

| **Variabele** | **Definition** |
| --- | --- |
| Author | First author as reported in paper. |
| Publication year | Year in which article was published in paper or online (if an electronic journal only). |
| Design | Retrospective: if patients are included based on a sample of patients is taken for whom index and readmission are already in the past.  Cross-sectional: if patients are included at the time point of readmission for whom only the index admission is in the past.  Prospective: if patients are included at discharge of index admission, irrespective whether they will or will not be readmitted.  If this cannot be clearly distinguished than the 3th reviewer decides which category suits best. |
| Country | Country in which the patient were recruited. In case of several countries than refer to the region (EU, western world, etc). |
| Setting | Monocenter: If patients are recruited in one hospital (irrespective whether this hospital has several locations); otherwise: multicenter. |
| All-cause | All-cause = if patients are selected at index AND readmission at ALL department; including if index AND readmission are not specified/ reported suggesting an all-cause admission. |
| Planned readmissions | Planned readmissions excluded = excluded before preventability was assessed  OR  Planned readmissions included if included but further no guidelines on how to review the planned readmissions  OR  Planned readmissions included and assigned to unavoidable category or a cause classification category.  OR  Not reported if it is not reported whether planned readmissions are included or not in the method section. |
| Reviewed cases* | Number of included patients for whom preventability of a readmission was assessed  OR  Number of included readmissions for which preventability was assessed.  *If possible, we calculated the number based on 30-day readmissions, in case readmissions with a longer (or shorter) time frame were included.  *if possible, we excluded planned readmissions. |
| Preventable readmissions | Number of included patients with a readmissions which was assessed as preventable  OR  Number of included readmissions which were assessed as preventable.  *If possible, we calculated the number based on 30-day readmissions, in case readmissions with a longer (or shorter) time frame were included.  *if possible, we excluded planned readmissions.  *Number calculated for reviewed readmission only.  *Some studies reported also possibly or potentially preventable readmissions and preventable readmissions. We included the number of preventable readmission as reported in the final analysis of the included study; or if it was not specified whether possibly or potentially preventable readmissions were included in the final analysis as preventable than we based the number only on preventable readmissions (thus excluding potentially or possibly preventable readmissions). |
| Duration | Time between index and readmission which was used as selection criteria divided in to 30 days or less OR more than 30 days. |
| Age | Average (mean or median) age in years for total readmission population (or total population if otherwise not provided) divided into: 70 years or older, 19-69 years or <18 years.  *If provided for categories only, calculate average and report that. |
| Gender | % of males in total readmission population (or total population if otherwise not provided)  *If provided for categories only, calculate average and report that. |
| Preventability definition | See supplementary file 4. |
| Score | Score used to assess preventability divided into: binary, category, scale or unknown (not reported, not specified, not applicable). |
| A priori | A priori cause classification= certain causes were a priori defined as ALWAYS preventable.  If not, than it is not an a priori cause classification. |
| Reviewers | Individual= reviewers individually assessed preventability  Duo= reviewers assessed preventability in dialogue with each other  Panel= all or a selection of reviews was discussed by a panel (>2 reviewers) to assess final preventability. |
| Resolvement | Single reviewer, second/third reviewer or majority agreement to assess final preventability. |
| Training | Were reviewers trained prior to start or during pilot phase? And or was a logbook or well defined protocol available? |
| Interview | Was interview (questionnaire, survey, interview) with patient (and/or family), discharging team or member of discharging team and/or general practitioner conducted in addition to medical file review only. |
