# Supplementary material for: How do studies assess the preventability of readmissions? A systematic review with narrative synthesis
Source: BMC Med Res Methodol. 2019 Jun 19;19:128. doi: 10.1186/s12874-019-0766-0 (PMC6585018; doi:10.1186/s12874-019-0766-0)
Supplement: Supplementary file 4 — Characteristics of studies which were excluded based on the inclusion criteria of the flow chart (N=29). (DOCX 24 kb) [file 12874_2019_766_MOESM4_ESM.docx]

| Author | Year | Country | Study design | Setting | Disease group/department  of index admission | Duration between index- and readmission | Reason for exclusion^1^ |
| --- | --- | --- | --- | --- | --- | --- | --- |
| Allen-liles | 2015 | USA | Prospective | monocenter | All departments | 30 days | 3 |
| Amin | 2016 | USA | Prospective | monocenter | Paediatrics | 30 days | 6 and 9 |
| Bell | 2015 | USA | Retrospective | monocenter | Internal medicine | 7, 15 and 30 days | 6 and 9 |
| Borzecki | 2015 | USA | Retrospective | monocenter | Pneumonia | 30 days | 3 |
| Brook | 2014 | USA | Retrospective | monocenter | Gastrointestinal cancer | 30 days | 4 |
| Cooksley | 2015 | UK | Retrospective | monocenter | Oncology | 30 days | 6, 7 and 8 |
| El-Jawahiri | 2016 | USA | Retrospective | multicenter | Acute myeloid leukaemia | 30 days | 3 |
| Experton | 1999 | USA | Prospective | multicenter | Frail elderly | 90 days | 9 |
| Gilotra | 2016 | USA | Prospective | monocenter | Heart failure | 30 and 60 days | 9 |
| Graham | 1983 | UK | Retrospective | monocenter | Geriatrics | 1 year | 6 and 7 |
| Haines-Wood | 1996 | Ireland | Prospective | monocenter | Elderly patients | 30, 90 and 180 days | 7 |
| Halfon | 2006 | Switzerland | Retrospective | multicenter | All departments | 30 days | 2 |
| Hauviller | 2016 | France | Retrospective | monocenter | Patients >65 years and ADR | 1 year | 4 |
| Heckenbleikner | 2013 | USA | Retrospective | monocenter | Colon or rectal resections | 30 days | 6 and 7 |
| Herzig | 2015 | USA | Cross-sectional | multicenter | general medicine | 30 days | 2 |
| Jackson | 2014 | USA | Retrospective | multicenter | All departments | 30 days | 2 |
| Kirk | 2006 | UK | Retrospective | monocenter | All departments | 28 days | 6 and 9 |
| Lee | 2017 | UK | Prospective | multicenter | Surgical patients | 30 days | 6 and 8 |
| Levy | 2000 | UK | Prospective | monocenter | Medical emergency patients | 28 days | 6 and 7 |
| Ludke | 1990 | USA | Retrospective | monocenter | All departments | 14 days | 3 |
| McIntyre | 2016 | USA | Retrospective | monocenter | General surgery | 30 days | 3 |
| Patel | 2016 | USA | Cross-sectional | monocenter | General medicine | NR | 4 |
| Phelan | 2009 | Ireland | Retrospective | monocenter | Heart failure | 1 year | 3 |
| Ruiz | 2008 | Spain | Prospective | monocenter | Adverse drug reactions | 60 days | 6 and 9 |
| Sutton | 2002 | UK | Retrospective | multicenter | Surgical department | 28 days | 6 and 9 |
| Trickey | 2016 | US | Retrospective | monocenter | Surgical patients | 30 days | 6 and 9 |
| Volk | 2012 | USA | Retrospective | monocenter | Decompensated cirrhosis | 30 days | 6 and 9 |
| Witherington | 2008 | UK | Retrospective | monocenter | Elderly patients | 28 days | 3 |
| Yap  *^1^Reason of exclusion = number corresponds with box in inclusion criteria flow chart, see supplement file 2* | 2016 | USA | Retrospective | monocenter | Pneumonia, septicemia or respiratory failure | 30 days | 6 and 9 |

Additional file **file 4: Characteristics of studies which were excluded based on the inclusion criteria of the flow chart (N=29)**
