# Supplementary material for: How do studies assess the preventability of readmissions? A systematic review with narrative synthesis
Source: BMC Med Res Methodol. 2019 Jun 19;19:128. doi: 10.1186/s12874-019-0766-0 (PMC6585018; doi:10.1186/s12874-019-0766-0)
Supplement: Supplementary file 5 — Detailed descriptives of included studies (N=48). (DOCX 33 kb) [file 12874_2019_766_MOESM5_ESM.docx]

| Author  Additional file **file 5: Detailed descriptives of included studies (N=48)** | Year | Country | Study design | Setting | Disease group/department of index admission | Duration between index- and readmission | Age (mean)^1^ | Male (%)^2^ |
| --- | --- | --- | --- | --- | --- | --- | --- | --- |
| Agrawal | 2015 | USA | Retrospective | Single center | Decompensated cirrhosis | 30 days | 59 | 100 |
| Auerbach | 2016 | USA | Cross-sectional | multicenter | General medicine | 30 days | 55 | NR |
| Balla | 2008 | Israel | Cross-sectional | single center | Medicine | 30 days | 70 | 56 |
| Bianco | 2012 | Italy | Cross-sectional | single center | Medical or surgical illness | 30 days | 64 | 54 |
| Burke | 2016 | USA | Retrospective | single center | Internal medicine | 7 and 30 days | 56 | 65 |
| Cakir | 2010 | USA | Retrospective | single center | Not specified | 30 days | 58 | 47 |
| Clarke | 1990 | UK | Retrospective | multicenter | General medicine/  surgery, geriatrics | 7 and 28 days | NR | NR |
| Dawes | 2014 | USA | Retrospective | single center | General surgery | 30 days | 55 | 51 |
| Epstein | 2014 | USA | Retrospective | single center | GI medical oncology | 7 and 30 days | 63 | 57 |
| Feigenbaum | 2012 | USA | Cross-sectional | multicenter | Not specified | 30 days | 71 | 45 |
| Fluitman | 2015 | Netherlands | Retrospective | single center | Internal medicine | 30 days | 71 | 38 |
| Frankl | 1991 | USA | Prospective | single center | Internal medicine | 30 days | 53 | 51 |
| Gautam | 1996 | Scotland | Prospective | single center | Geriatrics | 28 days | 81 | 42 |
| Glass | 2013 | USA | Retrospective | multicenter | Patients undergoing pancreatectomy | 30 days | NR | NR |
| Greenberg | 2016 | USA | Retrospective | single center | Patient with a aneurysmal subarachnoid haemorrhage | 30 days | 56 | 24 |
| Hain | 2016 | USA | Retrospective | single center | Paediatrics | 15 days | NR | 58 |
| Halfon | 2002 | Switzerland | Prospective | single center | All departments | 30 and 1 year | 53 | 57 |
| Haray | 2013 | USA | Retrospective | single center | Kidney transplantation | 30 days | 51 | 61 |
| Jiminez-Puente | 2004 | Spain | Cross-sectional | single center | All departments | 1, 3 and 6 mo | 54 | 58 |
| Jonas | 2016 | USA | Retrospective | single center | Paediatrics | 15 days | <18 | NR |
| Kelly | 2015 | UK | Retrospective | single center | Learning disability | 30 days | 45 | 56 |
| Koekkoek | 2011 | USA | Retrospective | multicenter | All departments | 21 days | 75 | NR |
| Maurer | 2004 | Switzerland | Prospective | single center | Internal medicine | 30 and 90 days | 65 | 53 |
| Meisenberg | 2016 | USA | Retrospective | single center | Oncology | 30 days | NR | NR |
| Miles | 1999 | USA | Retrospective | single center | All departments | 28 days | 44 | NR |
| Mittal | 2016 | USA | Retrospective | multicenter | Acute ischemic stroke | 30 days | 77 | 29 |
| Nahab | 2012 | USA | Retrospective | single center | Stroke and CVA | 30 days | 62 | 46 |
| Nijhawan | 2015 | USA | Retrospective | single center | HIV patients | 30 days | 43 | 66 |
| Njeim | 2012 | USA | Cross-sectional | single center | Internal medicine | 30 days | NR | NR |
| Oddone | 1996 | USA | Prospective | multicenter | General medicine | 6 months | 66 | 99 |
| Pace | 2014 | Canada | Prospective | multicenter | Medical wards | 30 days | 57 | 54 |
| Ryan | 2014 | USA | Retrospective | single center | Heart failure | 30 days | 81 | 35 |
| Saunders | 2015 | USA | Retrospective | single center | Oncology | 30 days | 69 | 45 |
| Shah | 2013 | UK | Retrospective | single center | Neurosurgery | 30 days | 55 | 53 |
| Shalchi | 2009 | UK | Retrospective | single center | Acute medical unit | 14 days | 75 | 57 |
| Shimizu | 2014 | USA | Cross-sectional | single center | Internal medicine | 30 days | 53 | 59 |
| Stein | 2016 | USA | Cross-sectional | single center | Internal medicine | 30 days | 53 | 50 |
| Sutherland | 2016 | UK | Retrospective | single center | Colorectal Surgery | 30 days | 54 | NR |
| Tejedor-Sojo | 2015 | USA | Retrospective | single center | Paediatrics | 30 days | <18 | 57 |
| Toomey | 2016 | USA | Cross-sectional | single center | Paediatrics | 30 days | <18 | 54 |
| Vachon | 2012 | USA | Retrospective | single center | Trauma patients | NR | 47 | 67 |
| Van Walraven | 2011 | Canada | Prospective | multicenter | Medicine and surgery | 6 months | 65 | 47 |
| Vinson | 1990 | USA | Prospective | single center | Congestive heart failure | 90 days | 80 | 45 |
| Wallace | 2015 | USA | Retrospective | single center | Paediatrics | 30 days | <18 | 59 |
| Wasfy | 2014 | USA | Retrospective | multicenter | PCI patients | 30 days | 67 | 64 |
| Weinberg | 2016 | USA | Retrospective | single center | Total hip arthroplasty | 30 and 90 days | 64 | 32 |
| Williams | 1988 | UK | Cross-sectional | single center | Geriatrics/ all departments | 28 days | >65 | 36 |
| Yam | 2010 | China | Retrospective | multicenter | Medicine | 30 days | 75 | 53 |

*^1^ If provided for categories only, the average was calculated*

*^2^NR=not reported*

*CVA= cerebrovascular event; PCI= percutaneous coronary intervention*
