# Supplementary material for: How do studies assess the preventability of readmissions? A systematic review with narrative synthesis
Source: BMC Med Res Methodol. 2019 Jun 19;19:128. doi: 10.1186/s12874-019-0766-0 (PMC6585018; doi:10.1186/s12874-019-0766-0)
Supplement: Supplementary file 6 — Details regarding patient (and/or caregiver) interview and care provider interview. (DOCX 40 kb) [file 12874_2019_766_MOESM6_ESM.docx]

|  |  | Patient interview | | | | |
| --- | --- | --- | --- | --- | --- | --- |
| **Additional file 6: Details regarding patient (and/or caregiver) interview and care provider interview** | **No^1^** | **interviewer** | **Interviewee** | **Format** | **Preventability assessment** | **Domains/ themes addressed** |
| Auerbach | 1000 | Trained assistants | Patient | Open- and closed-end questions | Available for reviewers | Social support, communication, follow-up appointment, management of medication, symptoms and appointments, recovery problems, thoughts on what could have helped avoid a hospital readmission.^1^ |
| Balla | NS^2^ | Author | Patient | NS | NS | Demographic data and clinical information pertaining to both admissions. |
| Bianco | NS^2^ | Trained physicians | Patient | NS | NS | NS |
| Burke | 6 | Author | Patient | NS | NA^3^ | NS |
| Feigenbaum | 390 | Nurses | Patient/caregiver | Open-ended and closed-ended | Available for reviewers | Medication changes, coordination of care, patient education, follow-up and potentially preventability of readmission. |
| Shimizu | 72 | NS | Patient | NS | NS | Self-identified ethnicity, primary language, living conditions, perceived medical understanding and barriers to care. Health literacy based on highest achieved education and 3-question screening survey. |
| Stein | 23 | Inpatient providers (IA) | Patient | Standardized form | Included | Adapted from Institute of Healthcare improvement (IHI) form; including: ’in your opinion, was this readmission preventable?’ |
| Sutherland | 48 | Surgical resident | Patient/caretaker | NS | Available for reviewers | Follow-up appointment, self-care problems, discharge instructions, any additional problems that led up to the readmission and would prevent future readmission. |
| Toomey | 1192 | Nurses or research assistant | Parent/caregiver | semi structured | included | NS |
| Van Walraven | 4812 | Study personnel | Patient | NS | NS | Baseline functional status and chronic medical conditions. |
| Vinson | NS | NS | Patient/family | NS | Available for reviewers | NS |
| Williams | 213 | NS | Patient/carer (n=190) | NS | NS | NS |
|  |  |  |  |  |  |  |
|  |  | **Physician interview** |  |  |  |  |
|  | **No^1^** | **interviewer** | **Interviewee** | **Format** | **Preventability assessment** | **Domains/ themes addressed** |
| Auerbach | 1785 | - | GP, IA and RA physician | Survey | Available for reviewers | Factors contributing to readmission and improvement opportunities (e.g. communication and discharge plans) |
| Feigenbaum | 538 | Nurses | GP, specialists, hospitalists, skilled burse facility physician and other physicians | Open-ended and closed-ended | Available for reviewers | Preventability and predictability of readmission, awareness of worsening patient condition, patient needs for advance care planning and possibility of providing care in nonhospital setting |
| Gautam | 109 | - | GP (96% response) and discharging consultant (99% response) | NS | Equal weighing of opinion interviewee and audit team | Avoidability of the readmission |
| Sutherland | NS | Surgical resident | Attending surgeon | NS | included | Readmission preventability factors that contributed to the readmission and the surgeon’s role in patient’s care. |
| Toomey | 267 | Paediatrician | GP | structured | NS | NS |
| Williams | NS^4^ | NS | Hospital ward sister and GP (n=99 with 212 responses) | NS | NS | NS |
|  |  |  |  |  |  |  |

*No=number; IA= index admission; GP= general practitioner; RA=readmission*

*^1^including 3-item care transition measure and interpersonal process of care measure; ^2^ reported was that all readmitted patients were interviewed. ^3^only interviews conducted during the pilot phase; ^4^ all but one ward sister was interviewed.*
