# Supplementary material for: How do studies assess the preventability of readmissions? A systematic review with narrative synthesis
Source: BMC Med Res Methodol. 2019 Jun 19;19:128. doi: 10.1186/s12874-019-0766-0 (PMC6585018; doi:10.1186/s12874-019-0766-0)
Supplement: Supplementary file 7 — Definition of preventability. (DOCX 24 kb) [file 12874_2019_766_MOESM7_ESM.docx]

**Additional file 7: Definition of preventability**

Sentences or description (with or without an additional set of criteria and/or cause classification) to explain how preventability or avoidability was defined in the method section of the articles, if applicable.

| **First author** | **Definition** |
| --- | --- |
| Agrawal | A readmission was considered potentially preventable if it was believed that it could have been prevented by one or more of the following: adequate-quality care during index hospitalization, an adequate medication reconciliation process at discharge, adequate postdischarge follow-up, or adequate coordination between the inpatient and outpatient healthcare teams. |
| Auerbach | In assessing preventability, we trained case reviewers to consider patient illness but to primarily focus on system flaws and gaps in care that could have been avoided with reasonable patient or physician activities. As a framing example, we trained physician adjudicators to consider an “ideal health system” as a model for system and care assessment, even if all aspects of an ideal system did not exist at their site. |
| Balla | The index admission was carefully evaluated for possible QOC (Quality Of Care) problems that were identified and classified according to a predetermined list; Once a QOC problem was identified, the clinician determined if the problem was preventable (avoidable) or not. |
| Bianco | Each readmission was classified as potentially avoidable or unavoidable by taking into account diagnostic and therapeutic process of the first admission. A researcher and an experienced physician concurrently used a set of criteria derived from previously published research. |
| Burke | We considered readmission attributable to provider error (categories 1 and 3) to be avoidable.^1^ |
| Cakir | Readmission was reported to be preventable if the medication reconciliation documentation at discharge was not done correctly, a vital test which could have changed the treatment was ordered as an outpatient following discharge, or if the patient was not instructed to see his primary care physician or treating specialist for follow up. |
| Clarke | The copies (case notes) were circulated to teams of assessors, who were asked to assess the avoidability of the second admission with respect to the hospital care given in the first admission. They were asked to classify the readmissions as avoidable, unavoidable, and unclassifiable according to a classification scheme provided to help their decisions. |
| Dawes | Readmissions were considered preventable if they fell into any of 3 predefined categories based on targets of improvement: premature hospital discharge, inadequate follow-up, and potential outpatient management 1: premature hospital discharge, 2. inadequate follow up and 3.potential outpatient follow up. Readmissions were considered preventable if they fell into any of 3 predefined categories based on targets of improvement: premature hospital discharge, inadequate follow-up, and potential outpatient management. |
| Epstein | We defined a readmission as preventable if it likely would not have occurred had either the index admission been prolonged until achievement of a realistically anticipatable medical improvement, or a reasonably foreseeable and accomplishable change in the discharge plan of the index admission had been made. |
| Fluitman | A readmission was judged as potentially preventable if it could have been reasonably foreseen by a discharging physician and could reasonably have been prevented by any action undertaken by hospital staff or the patient. |
| Glass | to determine the root causes of the readmission, as well as whether the admission had been warranted or potentially avoidable, and where the failures  occurred in the discharge and disposition planning system. |
| Greenberg | A liberal definition was used to categorize a readmission as likely avoidable if any shortcoming was identified in clinical care that may have significantly contributed to readmission, regardless of whether other contributing factors may have been present. |
| Hain | The panel members were charged to rate the degree to which readmissions were preventable by changes in the inpatient care or discharge planning provided during the preceding (“index”) hospitalization. |
| Halfon | All readmissions linked to problematic discharge (Categories d through f of appendix A )^1^ were considered attributable to hospital care and consequently avoidable. Conversely, readmissions from Categories g through j (see Appendix A) were considered beyond the control of the hospital services, although it may be argued that certain causes are probably within partial control of the care team (e.g., readmissions resulting from patient behaviour, post discharge follow-up, or recurrence of an existing disorder). Readmissions caused by complications of  care (Categories a through c in Appendix A) were more difficult to categorize because of the necessity of assessing medical and nursing services for compliance with expected practice standards; these cases were classified as avoidable or not by the consensus of two experienced clinical physicians and external experts if necessary. An adverse event was judged avoidable if it resulted from the medical intervention rather than from the natural evolution of the pathological factors and if it was foreseeable and routinely preventable. |
| Jiminiez- Puente | Readmissions were classified according to the criteria shown in Table 1, into one of two possible groups: potentially avoidable or unavoidable, after analysis of the diagnostic therapeutic process in the previous admission and its possible  modification in each case. |
| Jonas | Because this analysis was done as part of a quality improvement initiative, it focuses on 15-day, “early readmissions” to target cases with a higher probability  of being potentially preventable from the perspective of the hospital care team. A diagram of the fault tree framework, color coded to indicate which nodes were considered potentially preventable, is shown in Figure 1. |
| Koekkoek | For ascertaining preventability, the reviewer decided whether a change in the discharge plan or immediate posthospitalization plan of care would have reduced the likelihood of readmission. |
| Ludke | Determinations of appropriateness were made solely on the basis of the clinically oriented ISD^1^ criteria and did not take into consideration factors such as physician convenience, availability of outpatient facilities, or nonclinical patient characteristics such as distance from the medical center. |
| Miles | Preventability was defined as ‘an error in management due to failure to follow accepted practice at an individual or system level’ and accepted practice was taken to be ‘the current level of expected performance for the average practitioner or system that manages the condition’. |
| Mittal | Further, a potentially preventable 30-d readmission was defined as any readmission from any cause which potentially could have been prevented by an intervention during the index hospitalization as agreed upon by two study neurologists |
| Nahab | We defined patients with avoidable readmissions as those patients whose primary diagnosis for readmission was considered the direct result of modifiable factors during the index hospitalization.  We reviewed all readmissions for elective procedures and considered them avoidable only when there was no medical indication to delay the procedure beyond the index hospitalization. |
| Oddone | For preventable admissions, they applied a checklist, developed by qualitative analytic methods, to define the specific resources or conditions that, if available before hospitalization, might have averted the patient's admission (e.g., readily available outpatient diagnostic tests). |
| Pace | The returns were classified as (1) preventable because of an error on discharge (such as with the medication prescription, or failure to arrange follow-up) or because of patient noncompliance (not attending follow-up, not taking medication appropriately, not following lifestyle modification suggestions); (2) potentially preventable because increased resources could have prevented return (eg, increased availability of services in the community. |
| Ryan | Providers were given no specific guidelines for deciding whether a readmission was preventable. This allowed use of their different backgrounds in choosing which elements of the clinical record to focus on. |
| Saunders | The primary purpose of this analysis was to define readmissions which, in the authors’ opinion, may have been prevented around the time of discharge. This would typically involve either choosing an alternate therapy for existing conditions, delaying discharge or improvements in the discharge process. |
| Shalchi | For each patient, each clinician gave an opinion as to whether they felt the readmission was in some way related to the index admission, as well as whether it could have been avoided with more judicious care. |
| Sutherland | In this analysis, a preventable re-admission was defined as, “one that could have been avoided had reasonable assessment, treatment, monitoring, access, and services been put in place and appropriate systems been available to support the patient’s care transition.” |
| Wallace | A preventable readmission was defined as one with either a caretaker- or physician related reason for readmission. Subjects with only disease-related or unrelated reasons for readmission were deemed to have a nonpreventable readmission. |
| Wasfy | For this analysis, we sought to identify readmissions that were “clinically preventable”—defined as those preventable by a change in clinical decision-making by a clinician under the current standards of care. |
| Weinberg | Potentially preventable readmissions therefore were defined as conditions or diagnoses that could have been avoided through some intervention during the index hospitalization based on criteria published elsewhere or practice guidelines. |
| Williams | It was noted that readmission could have been avoided if more effective action had been taken in one or more of five areas: preparation for and timing of discharge, attention to the needs of the carer, timely and adequate information to the general practitioner and subsequent action by the general practitioner, sufficient and prompt nursing and social services support, and management of medication. |
| Others | A subset of articles did not use a ‘sentence’ to define what they considered preventable, instead they used a different approach. Preventability based on a set of criteria (often causes, interventions , and sometimes combined with an a priori preventability qualification).   - Feigenbaum, Harhay, Kelly, Maurer, Meisenberg, Nijhawan, Shah, Shimizu, Tejedor, van Walraven, Vachon, Vinson, Yam; - Njeim, Stein, Toomey (State Action on Avoidable Rehospitalizations initiative) - Frankl, Gautam (Not a set of criteria but preventability assessed via individual and team approach , see table 1) |

^1^ See the original paper for more details on categories and/or abbreviations.
